# Chemical Transformations of Lignin Under the Action of 1-Butyl-3-Methylimidazolium Ionic Liquids: Covalent Bonding and the Role of Anion

**DOI:** 10.3390/ijms262311627

**Published:** 2025-11-30

**Authors:** Artyom V. Belesov, Ilya I. Pikovskoi, Anna V. Faleva, Dmitry S. Kosyakov

**Affiliations:** Core Facility Center ‘Arktika’, M.V. Lomonosov Northern (Arctic) Federal University, Arkhangelsk 163002, Russia; i.pikovskoj@narfu.ru (I.I.P.); a.bezumova@narfu.ru (A.V.F.)

**Keywords:** lignin, ionic liquids, 1-butyl-3-methylimidazolium, anion, transformation

## Abstract

1-Butyl-3-methylimidazolium (bmim) ionic liquids (ILs) are widely used for lignocellulose fractionation, yet their role extends beyond mere solvents. This study revealed that bmim-based ILs act as active chemical reagents, modifying the lignin structure in an anion-dependent manner. Thermal treatment (80–150 °C) of spruce dioxane lignin with [bmim]OAc, [bmim]Cl, and [bmim]MeSO_4_ resulted in two distinct transformation pathways. In [bmim]MeSO_4_, acidic catalysis dominates, leading to lignin condensation (increase in weight-average molecular weight, M_w_, to 15.2 kDa at 150 °C) and intense sulfur incorporation (up to 9.9%) via anion-derived methylation/sulfation. Conversely, [bmim]OAc promotes depolymerization (decrease in M_w_ to 3.6 kDa) and efficient covalent bonding of the bmim cation to lignin (up to 10.8 cations per 100 aromatic units and a 6.5% nitrogen content at 150 °C), preventing condensation. Two-dimensional NMR and HPLC-HRMS analyses revealed the formation of a C–C bond between the C_2_ atom of the imidazole ring and the α-carbon of the phenylpropane lignin fragments and allowed for the identification of a number of individual nitrogen-containing lignin oligomers in the [bmim]OAc-treated samples. Their formation likely proceeds via nucleophilic addition of the N-heterocyclic carbene (NHC), derived from the bmim cation by deprotonation with the highly basic acetate anion, to aldehyde groups. The action of [bmim]Cl primarily induces acid-catalyzed transformations of lignin with minimal covalent modification. These findings redefine imidazolium ILs as reactive media in biorefining, where their covalent interactions can influence the properties of lignin but complicate its native structure and the recyclability of the IL.

## 1. Introduction

Lignin, the second most abundant biopolymer on Earth, represents a vast renewable source of sustainable polymer materials and aromatic compounds for the chemical industry [1,2,3,4]. Its complex, three-dimensional, amorphous macromolecular structure is derived primarily from three hydroxycinnamyl alcohol monomers (*p*-coumaryl, coniferyl, and sinapyl alcohols) linked by a variety of C–O (e.g., β–O–4) and C–C bonds, forming a robust heteropolymer that is intrinsically resistant to degradation. However, its complex cross-linked structure, which is formed by robust C-C and C-O-C bonds and is involved in the formation of covalent bonds with the polysaccharide matrix, makes native lignin resistant to solubilization [5]. Consequently, conventional biomass fractionation technologies often employ harsh treatment conditions and aggressive media that either deeply degrade lignin or facilitate undesirable condensation processes, narrowing their application potential. The vast majority of technical lignin is currently burned as a low-value fuel or considered a waste in bioethanol and pulp and paper production. In this regard, the development of new biorefining technologies based on the integrated use of plant biomass and the production of lignin preparations suitable for processing into high-added-value products is of great importance [6].

In addition to some deep eutectic solvents, ionic liquids (ILs), particularly those based on the 1-butyl-3-methylimidazolium (bmim) cation, have emerged as promising media for the sustainable fractionation of lignocellulosic biomass [7,8]. Their unique properties allow for the complete dissolution of lignocellulose [9], efficient cleavage of lignin-carbohydrate and some inter-unit linkages [10,11], and easy solvent recyclability [12,13,14]. The possibility of selective precipitation of lignin- and polysaccharide-rich fractions from an IL solution of plant biomass [15] opens up prospects for the development of novel biorefining technologies that meet modern principles of green chemistry and a sustainable economy. One of the main products of such technologies is so-called IL-lignin, whose properties differ significantly from those of other known types of technical preparations of the biopolymer (soda, kraft, hydrolysis, and organosolv lignins) [16,17]. The chemical composition and structural features of IL-lignins determine their direction of use and are significantly influenced by biopolymer transformations during biomass dissolution in ILs, which requires elevated temperatures (80–150 °C) [18,19,20].

A pivotal study by Wen et al. [21] systematically investigated the chemical transformations of isolated (alkaline cooking) poplar lignin during treatment with 1-ethyl-3-methylimidazolium acetate across a range of temperatures and reaction times. The results clearly revealed changes in the hydroxyl group content (via dehydration) and S/G (syringyl/guaiacyl structures) ratio, cleavage of the β-O-4 ether and even degradation of the β-β′ and β-5′ carbon–carbon linkages in the lignin structure at high temperatures resulted in partial depolymerization and concomitant condensation reactions. Similar conclusions concerning the role of competing depolymerization (e.g., β-O-4 cleavage) and condensation (C-C bond formation) reactions in the changes in the macromolecular properties of various lignin preparations under the action of ILs have also been reached in other studies [22,23,24].

In recent years, a growing body of evidence has suggested that 1,3-dialkylimidazolium ILs act not only as solvents and inert media for the transformation of lignin but also as active chemical agents capable of interactions with the biopolymer [25]. Thus, an elemental analysis of lignins isolated from wood by dissolution in bmim-based ILs revealed substantial nitrogen content (up to several percent), suggesting the incorporation of the IL cation in the lignin structure [26]. Covalent bonding of ILs can occur through deprotonation of the cation (e.g., when interacting with a highly basic anion) with the formation of a highly reactive intermediate—N-heterocyclic carbene (NHC). The latter readily reacts with aldehyde groups via nucleophilic addition [27] and decomposes with the formation of various nitrogen-containing compounds, including those capable of further interactions with lignin [28]. Our recent study involving a number of monomeric lignin model compounds [29] unequivocally demonstrated the possibility of NHC addition to aldehyde and other electron-deficient functional groups in the lignin structure (ketone, aliphatic hydroxyl, and double carbon–carbon bonds in the propane side chains), resulting in a variety of nitrogen-containing reaction products.

Although experiments on model compounds provide crucial mechanistic insights, they cannot fully reproduce the complex and heterogeneous environment of the native biopolymer. The diversity of functional groups in its structure and competing side reactions can significantly alter the transformation pathways and resulting products [24,30,31]. However, despite the recent discovery of charged monomeric and oligomeric bmim adducts by MALDI mass spectrometry [32] and the observation of the signals of covalently bound bmim cations in the IL lignin NMR spectra [33], systematic studies of the formation of IL-modified lignin structures at the molecular level are still lacking in the literature. Moreover, the role of an IL anion, which is potentially capable of both influencing carbene formation and directly interacting with lignin, remains unclear [15,34,35]. This impedes the rational selection of ILs as biomass processing media for targeted lignin valorization and does not allow to conclude that covalent modification with ILs is an undesirable side reaction or a potential tool for creating tailored lignin-based materials.

The present study aims to close this gap by revealing the patterns of chemical transformation of native lignin during interactions with bmim-based ILs containing anions of various chemical natures under different conditions and reliably identifying the key covalent interaction products. To achieve these objectives, an approach based on prolonged (24 h) treatment of a softwood dioxane lignin preparation, which is close in structure to the native biopolymer, with bmim acetate ([bmim]OAc), methyl sulfate ([bmim]MeSO_4_), and chloride ([bmim]Cl) with varying temperatures in the range typical for biorefining processes (80–150 °C) was used. The choice of ionic liquids was determined by their successful application for the dissolution and fractionation of plant biomass [36,37,38]. Additionally, this set of ILs, with the common [bmim]^+^ cation and anions of varying basicity (OAc > Cl > MeSO_4_), provides a model system to isolate and study the specific role of the anion. Although deep eutectic solvents (DESs) offer certain practical advantages, their inherently complex and dynamic hydrogen-bonding networks, along with often ill-defined stoichiometries, introduce significant complications for pinpointing exact reaction mechanisms and attributing reactivity to specific chemical entities. Therefore, for this mechanistic investigation aimed at establishing clear principles of anion-driven reactivity, molecular ionic liquids present a superior and more rigorous experimental system. The structural changes in the resulting IL-lignin preparations were comprehensively characterized via size-exclusion chromatography, quantitative ^31^P and 2D NMR spectroscopy, elemental analysis, and high-performance liquid chromatography-high-resolution mass spectrometry (HPLC-HRMS) to correlate the IL composition and treatment conditions with specific modification pathways, including NHC-driven cation grafting and anion-derived functionalization.

## 2. Results

### 2.1. Molecular Weight Characteristics of IL-Treated Lignin

Analysis of the molecular weight distribution (MWD) via size-exclusion high-performance liquid chromatography (HPLC) (Supplementary Figure S1) revealed three distinct fractions in all of the modified samples. They form corresponding maxima on the MWD curve in the low (<1 kDa), medium (1–20 kDa), and high-molecular-weight (>20 kDa) regions. However, the dynamics of molecular weight changes differ drastically depending on the anion of the IL (Table 1).

Compared with the other studied ILs, treatment with [bmim]OAc resulted in a distinct predominance of the low-molecular-weight fraction in the obtained IL lignin. Notably, this effect increases with increasing treatment temperature. Thus, the proportion of the low-molecular-weight fraction increases from 7% to 13% when the temperature changes from 80 to 150 °C, clearly indicating the predominance of polymer chain scission reactions. Correspondingly, the weight-average molecular weight (M_w_) monotonically decreases from 7.7 kDa to 3.6 kDa, while the polydispersity index (PDI) also decreases from 5.1 to 3.0.

In contrast, the [bmim]MeSO_4_ treatment resulted in the opposite trend: intense condensation. In this case, the M_w_ increases with temperature, reaching a maximum of 15.2 kDa at 150 °C, and the proportion of the high-molecular-weight fraction doubled (from 7% to 14%) compared with that of the sample obtained at 80 °C. This evidence highlights the dominance of reactions leading to the formation of new interunit bonds and the enlargement of macromolecules.

In the case of [bmim]Cl, the changes in M_w_ were less pronounced. A slight initial increase in the molecular weight in the range of 80–100 °C followed by a decrease at 120–150 °C suggests a combination of moderate condensation and acid-catalyzed hydrolysis. The proportions of both the low-molecular-weight and high-molecular-weight fractions changed insignificantly. The only exception was a noticeable increase in the >20 kDa fraction after the IL treatment at 150 °C.

### 2.2. Changes in Functional and Elemental Composition

Hydroxyl and carboxyl groups are highly reactive and participate in the formation of bonds between lignin structural units. Therefore, monitoring changes in their content provides valuable information about ongoing structural transformations. Quantitative ^31^P NMR spectroscopy after derivatization with 2-chloro-4,4,5,5-tetramethyl-1,3,2-dioxophospholane, the most powerful technique for distinguishing -OH groups in lignin structures, allowed for the selective determination of phenolic (-OH_phen_) and aliphatic (-OH_aliph_) hydroxyl and carboxyl groups. The results (Table 2) revealed substantial changes associated with the treatment temperature and the nature of the ILs’ anions.

The most drastic changes occurred in the samples treated with [bmim]MeSO_4_. The total OH-group content decreased from 7.0% (80 °C) to 3.7% (150 °C), with reductions in both the aliphatic (from 3.8% to 1.3%) and phenolic (from 3.2% to 2.4%) hydroxyl contents. This is consistent with their assumed involvement in condensation and possibly esterification (e.g., sulfation) reactions.

Although treatment with [bmim]OAc also led to a decrease in the total OH-group content, the observed pattern was apparently different: while the number of aliphatic OH-groups decreased (from 6.4% to 3.5%), the content of -OH_phen_ increased from 3.5% to 5.1%. This pattern is typical for depolymerization processes where the cleavage of β-O-4 linkages releases new phenolic fragments. At temperatures above 120 °C, partial decarboxylation of lignin occurs, but the action of [bmim]OAc leads to the opposite effect: the content of COOH groups increases with temperature, which indicates the predominance of oxidation processes of aliphatic hydroxyl or aldehyde groups in this medium.

**Figure 1:**
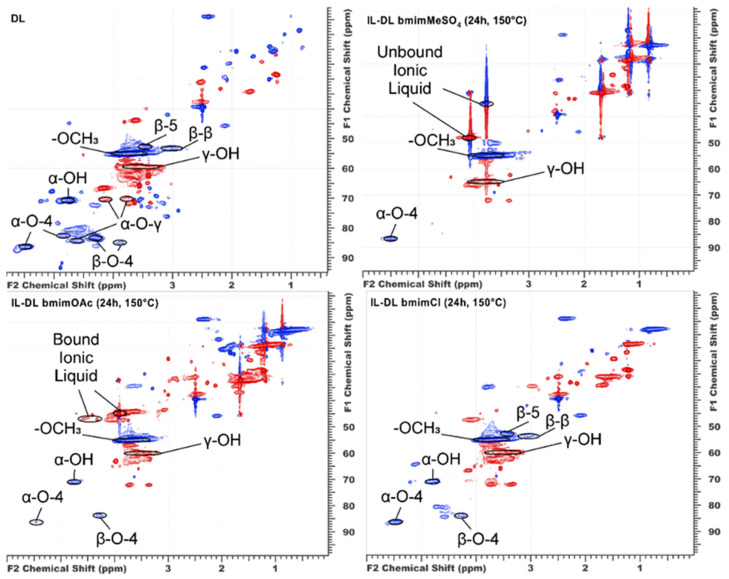
2D ^1^H–^13^C HSQC NMR spectra (aliphatic side chain region) of the parent DL and IL lignin preparations obtained by treatment at 150 °C.

Two-dimensional HSQC (Heteronuclear Single Quantum Coherence) NMR analysis on the ^1^H and ^13^C nuclei of the samples treated at 150 °C provided additional structural insights (Figure 1 and Figure S2) because other key functional groups (methoxyl and aldehyde) and interunit linkages, such as alkyl-aryl ether β-O-4 (the main and most labile type of bonds in the native lignin structure), alkyl-aryl ether α-O-4/carbon–carbon β-5 in the common phenylcoumarane dimeric structures, and alkyl-aryl ether α-O-γ/alkyl-alkyl β-β in pinoresinol-type dimeric structures, were determined.

Compared with the parent DL preparation, all of the studied IL-DL samples presented a dramatic decrease in the content of β-O-4 linkages (Table 3), confirming that the cleavage of alkyl–aryl ether bonds serves as a main pathway for lignin depolymerization in ILs. The maximum changes were observed for [bmim]MeSO_4_, whose interaction with lignin resulted in the complete disappearance of this linkage type. Moreover, the action of [bmim]MeSO_4_ was accompanied by an increase in the number of phenylcoumarane structures, which is evidence of ongoing condensation processes with the formation of alkyl–aryl carbon–carbon bonds. Notably, another specific feature of this IL is the methylation of lignin, which apparently involves the methylsulfate anion. Thus, while heating in other ILs results in some demethoxylation of lignin, the product obtained after [bmim]MeSO_4_ treatment contained 10% more methoxyl groups than the parent DL did.

Since carbonyl groups are capable of actively interacting with the deprotonated bmim cation (NHC), the change in their content during the treatment of lignin with ILs is of particular interest. Indeed, the number of -C=O groups in the IL-DL preparations was reduced by 70–100% compared with the initial DL. The corresponding signals completely disappeared in the NMR spectrum of lignin modified with [bmim]OAc.

Analysis of the aromatic region of the HSQC spectra (Figure S2) confirmed the softwood nature of the initial DL, showing a predominance of guaiacyl (G) units with a minor contribution of *p*-hydroxyphenyl (H) units. The G/H ratio remained largely unchanged after IL treatment, indicating the stability of the aromatic core under the conditions applied.

Elemental (CHNS) analysis confirmed the incorporation of the IL components into the obtained IL-DL preparations (Table 4). Treatment with [bmim]OAc led to a significant increase in the nitrogen content with increasing temperature, which changed from 2.4% at 80 °C to 6.5% at 150 °C. This indirectly indicates the covalent grafting of the bmim cation onto the lignin framework. In the case of [bmim]MeSO_4_, large-scale sulfur incorporation was also observed, with its content reaching 9.9% at 150 °C, indicating reactions between lignin and the methyl sulfate anion with the formation of sulfate or methylsulfate esters (Supplementary Figure S3). The maximum nitrogen content in this case was moderate (4.7%), suggesting a lesser role for the interactions with bmim cations. For [bmim]Cl, the nitrogen content remained low (~1%) regardless of the treatment temperature.

Apparently, a significant portion of the detected nitrogen may be associated with the presence of difficult-to-remove residues of free IL, which can be bound by lignin through donor–acceptor interactions or by an ion-exchange mechanism.

The covalent bonding of bmim was confirmed by the intense signals of the bmim-C_aliph_-lignin fragment in the HSQC NMR spectrum at δ 47.2/3.9 ppm (Figure 1). In the cases of bmim chloride and methylsulfate, the corresponding cross-peaks were less pronounced and overlapped with the signals of the unbound IL residues and other structures. The quantitative assessment of the bound bmim in the IL-DL preparation obtained at different temperatures via ^1^H-^13^C HSQC NMR (Table 5) revealed a fundamental difference between the studied ILs. In [bmim]OAc, the fraction of the covalently bound bmim cation increased monotonically with treatment temperature, reaching 10.8 per 100 aromatic units at 150 °C. In contrast, in [bmim]MeSO_4,_ the amount of bound cations was negligible (0.5 units/100 Ar), and the largest portion of the IL was present in an unbound form, suggesting a fundamentally different interaction mechanism.

### 2.3. Molecular-Level Identification of Nitrogen-Containing Compounds

The most reliable evidence of covalent bonding of the IL cation by lignin is the identification of the corresponding structures at the molecular level via high-resolution mass spectrometry. Since electrospray ionization in positive ion mode (ESI+) has a certain selectivity toward nitrogen-containing compounds because of their tendency to protonate in solution, as well as the possibility of forming structures with a fixed charge (bound cation bmim), its use allows us to obtain a visual representation of the presence of corresponding structures in lignin. For such studies, the IL-DL preparation obtained using [bmim]OAc at 150 °C was used because of its high nitrogen content and, as a consequence, simplification of the detection of oligomers covalently modified with an IL cation. The direct injection of the methanol-soluble fraction into an ESI ion source of an Orbitrap mass spectrometer allowed recording of the mass spectrum (Supplementary Figure S4) in the *m*/*z* range of 100–1000, which was dominated by the peaks of nitrogen-containing lignin compounds (mono-, di-, tri-, and tetrameric) according to their accurate mass-based elemental compositions. All of them contained two nitrogen atoms (as in bmim), and the gross formula of the largest detected oligomer (tetramer) ion was [C_47_H_55_O_12_N_2_]^+^ (*m*/*z* 839.3768, Δ = 2.1 ppm). The total number of detected nitrogen-containing compounds exceeded 400.

Direct-injection lignin mass spectra are distinguished with high complexity and contain signals of many isomeric and isobaric compounds at each nominal mass [39]. Their presence within the isolation window of the quadrupole mass filter (>0.4 Da) prevents the acquisition of tandem mass spectra of individual compounds and, consequently, hinders structure elucidation. To overcome this problem, reverse-phase chromatographic separation was used, which significantly improved the purity of the resulting mass spectra in the mass range of up to 500 Da.

**Figure 2:**
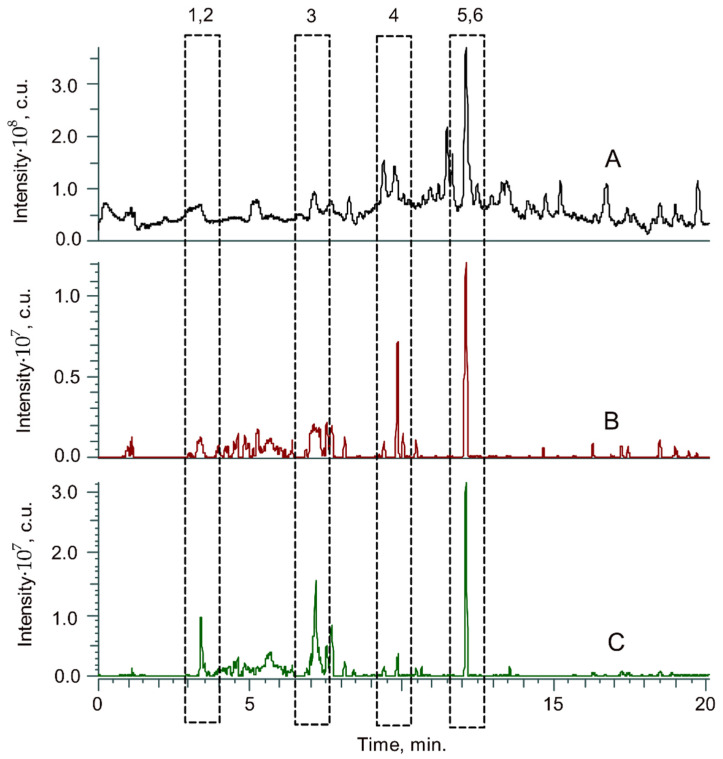
TIC (**A**) and XPIC at *m*/*z* 139.1229 (**B**) and 137.1073 (**C**) HPLC–HRMS chromatograms of the IL-DL sample obtained by treatment with ([bmim]OAc at 150 °C).

**Figure 3:**
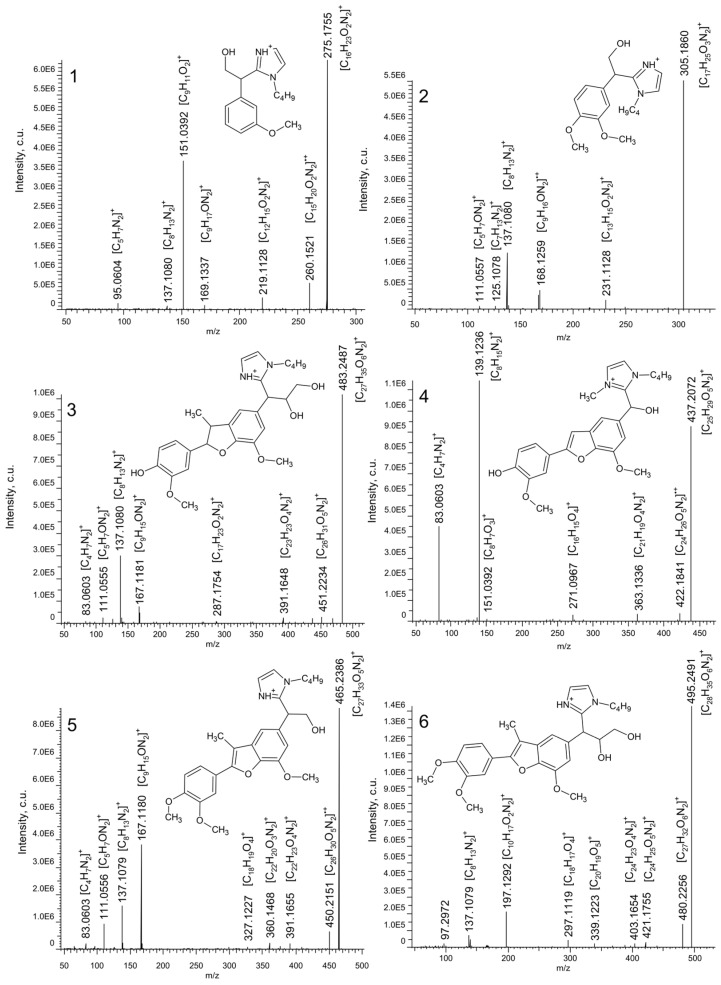
Tandem (HCD MS/MS) mass spectra and tentative identification of nitrogen-containing lignin compounds in the IL-DL preparation obtained by treatment with [bmim]OAc at 150 °C. The compound numbers correspond to those in Figure 2 and Table 6.

As expected, the obtained total-ion current (TIC) chromatogram (Figure 2A) contained an extremely large number of peaks, which complicates the nontargeted search for the compounds of interest. For this reason, an analytical strategy involving a target search of the bmim signals in tandem (MS/MS) mass spectra was implemented. The latter were the product ions with *m*/*z* 139.1229 ([C_8_H_15_N_2_]^+^) and 137.1073), corresponding to bmim and 1-butyl-2-methylimidazole moieties, respectively, which were eliminated from the bmim-modified lignin oligomers during collision-induced dissociation and served as a signature of the bmim moiety [32,33]. The corresponding accurate mass-extracted product ion current (XPIC) chromatograms (Figure 2B,C) demonstrate a number of intense peaks with retention times coinciding with both major and minor peaks on the TIC. The identification of the corresponding precursor ions (Table 6), followed by the acquisition of their tandem mass spectra (Figure 3), provided in-depth structural information.

Six major nitrogen-containing compounds were reliably identified (Table 6). Their RDB (ring and double bond equivalent) values ranging from 6.5 to 12.5 (the fractional values are typical for ions) are consistent with the formation of structures containing one to two aromatic lignin units (RDB = 4) covalently linked to a bmim (RDB = 2.5) fragment, providing direct proof of the proposed grafting mechanism. The presence of two additional RDB units in dimeric compounds with RDB = 11.5 and 12.5 likely indicates the formation of an additional ring with or without a double bond, which is characteristic of phenylcoumarane structures. Interpretation of the tandem mass spectra (Figure 3) allowed us to propose tentative structural formulas for the identified compounds. The fragmentation patterns of compounds 1 and 2 were dominated by neutral losses characteristic of the 1-butylimidazolium chain (e.g., M–C_4_H_8_) and the presence of the key 1-butylimidazolium fragment at *m*/*z* 137.1073, alongside ions derived from lignin-like aromatic units (e.g., *m*/*z* 151.0392). These data suggest that the structures of *1* and *2* are 2-butylimidazole-2-(3-methoxyphenyl)ethanol and 2-(3,4-dimethoxyphenyl)-2-butylimidazole-ethanol, respectively, which are likely formed by the addition of NHC to monomeric lignin degradation products.

During fragmentation of the higher-molecular-weight compounds *4*, *5*, and *6*, the charge was predominantly retained on the imidazolium fragment. The MS/MS spectra also revealed signature fragment ions with the following elemental compositions: [C_16_H_15_O_4_]^+^ (*m*/*z* 71.0967), [C_18_H_19_O_4_]^+^ (*m*/*z* 299.1280), and [C_20_H_19_O_5_]^+^ (*m*/*z* 339.1223). While these exact structures cannot be unambiguously assigned from MS data alone, the elemental compositions of the product ions and the supporting 2D NMR spectroscopy data indicated the presence of phenylcoumarone structures. It has been proposed that compounds *4-6* are adducts formed by the addition of NHC to phenylcoumarone-type structures, specifically, 1-butyl-3-methylimidazole-(CH-OH)-phenylcoumarone (*4*), 1-butylimidazole-(CH-CH_2_-OH)-4-methoxyphenylcoumarone (*5*), and 1-butylimidazole-(CH-1,2-ethan-2-ol)-4-methoxyphenylcoumarone (*6*). Similarly, the mass spectrum of compound *3,* with an RDB of 11.5, suggests the attachment of the 1-butylimidazolium fragment to a phenylcoumaran structure, leading to the proposed identification as 1-butylimidazole-(CH-1,2-ethan-2-ol)-phenylcoumarane. Notably, in most described lignin–IL interaction products, the bmim moiety is in the demethylated form (1-butylimidazolium). This fact is consistent with the observations made for monomeric model lignin compounds [29] and is explained by the partial degradation of the IL. The coherent identification of these specific structural motifs, which correlate MS data with NMR findings, provides compelling evidence for the covalent grafting of the bmim cation onto a diverse range of lignin-derived fragments and oligomers.

## 3. Discussion

### 3.1. IL Anion-Governed Dichotomy of Lignin Transformation Pathways

The obtained results clearly demonstrate that ILs cause substantial shifts in molecular mass distribution via depolymerization/condensation processes, dramatic changes in elemental and functional composition, and direct modification of the biopolymer via covalent bonding of ILs. The pathways and mechanisms of such transformations strongly depend on the nature of the anion of the IL, revealing a clear dichotomy between [bmim]OAc and the two other studied ILs.

Apparently, this difference is associated with the basicity of the anion, which determines the protolytic equilibrium of the solution. Unlike chloride and methylsulfate, acetate anions, which have a relatively high affinity for protons, create a basic environment in aqueous solutions. Despite the specific environments in nonaqueous ILs, a large difference in the Brønsted basicity between acetate and other studied anions remains, which is experimentally confirmed by ^13^C and ^15^N NMR using 1,3-dimethyl-2-imidazolidinone as a relevant probe prone to protonation [40]. Furthermore, even trace amounts of water, which is inevitably contained in hydrophilic ILs, may also participate in acid–base equilibria with ILs and thus contribute to hydrolytic degradation of lignin or condensation reactions. The decrease in molecular weight and increase in phenolic OH-group content with increasing treatment temperature (Table 1 and Table 2) in the case of [bmim]OAc unequivocally point to the predominance of depolymerization reactions involving the destruction of β-O-4 linkages, similar to alkaline hydrolysis.

In contrast, as the conjugated form of a strong acid, the methyl sulfate anion creates a less basic or even acidic environment. Under these conditions, protonation of the lignin side chain or elimination of water molecules leads to the generation of highly reactive carbocations. These carbocations readily attack the electron-rich aromatic rings of other lignin fragments, resulting in condensation reactions and a sharp increase in molecular weight (Table 1). Concurrently, the anion itself acts as an alkylating and sulfating agent, as confirmed by the significant increase in methoxyl group content (Table 3) and the extremely high incorporation of sulfur into the lignin structure (Table 4).

An IL with a chloride anion occupies an intermediate position in this system. The chloride anion is a weak Lewis acid, leading to acid-catalyzed transformations similar to but less pronounced than those with [bmim]MeSO_4_. The absence of either strong basicity or alkylating/sulfating capability results in minimal covalent binding of the IL components to lignin.

### 3.2. Mechanism of Covalent Cation Bonding: The Role of N-Heterocyclic Carbene

**Figure 4:**
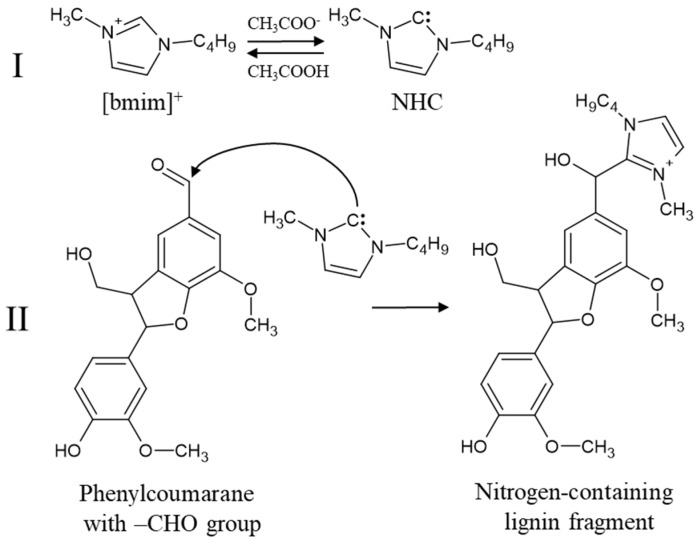
Covalent bonding of the bmim cation to the phenylcoumarane structure with the stages of NHC formation under the action of the acetate anion (**I**) and the addition of NHC to the aldehyde group (**II**).

The most important property of highly basic ILs is their ability to activate bmim cations via proton transfer, resulting in the formation of NHCs. Although NHC was not directly detected in this study, its role as the key intermediate is strongly supported by multiple lines of evidence. First, the presence of NHC in [bmim]OAc under similar conditions has been unequivocally demonstrated by Chiarotto I. et al. [27]. Our data are completely consistent with the results obtained previously for model compounds [29] and provide evidence that NHC is a key agent responsible for covalent lignin modification in [bmim]OAc (Figure 4).

First, the complete disappearance of aldehyde groups in the [bmim]OAc-treated samples (Table 3) is a classic marker of a nucleophilic addition reaction. NHC, a powerful nucleophile, efficiently attacks the carbonyl carbon of the aldehyde and ketone groups of lignin. This mechanism is corroborated by experiments with model phenols, where cross-peaks corresponding to the formation of a C–C bond between the C_2_ atom of the imidazole ring and the α-carbon of lignin fragments were detected.

Second, the observed effective suppression of condensation at high temperatures (low M_w_) is directly linked to this “capping” process. The reactive fragments generated during depolymerization (such as aldehydes), instead of undergoing recondensation reactions, become covalently bound to the bulky and stabilized bmim cation, which sterically and electronically hinders their further interaction.

Third, the HPLC-HRMS data (Table 6) provide direct evidence of the formation of hybrid molecules. The identified compounds with high accuracy correspond to structures where a bmim fragment is covalently attached to mono- and dimeric lignin units. This finding definitively confirms that the high nitrogen content (Table 4) and significant amount of covalently bound cations (Table 5) are the result of the targeted chemical synthesis of new compounds rather than physical contamination.

Our findings on the anion-dependent dichotomy align with and significantly extend the literature. For example, the role of basic anions in promoting depolymerization via β-O-4 cleavage is consistent with observations by Wen et al. [21], while we provide direct molecular evidence for the concurrent ‘capping’ mechanism that suppresses condensation. Similarly, the profound sulfur incorporation and methylation we observed with [bmim]MeSO_4_ go beyond the general reports of acid-catalyzed condensation [23], revealing specific covalent functionalization by the anion. The direct identification of bmim-lignin oligomers via HPLC-HRMS, as achieved here, provides a level of mechanistic insight that was previously inferred from elemental analysis [26] or NMR [34].

### 3.3. Competing Processes in the [bmim]MeSO4 System: Condensation vs. Functionalization

The transformation of lignin in [bmim]MeSO_4_ represents complex competition between two parallel processes driven by the acidity/basicity and nucleophilicity of the anion. The dominant process is acid-catalyzed condensation, as clearly demonstrated by the monotonic increase in lignin molecular weight (Table 1) with temperature. The formation of new C–C bonds between lignin fragments results in a more condensed and chemically stable polymer.

Simultaneously, anion-driven functionalization occurs. The sharp decrease in total OH- group content, particularly that of aliphatic OH (Table 2), combined with high sulfur incorporation (Table 4) convincingly indicates sulfation reactions. Shifts in the cross-peaks in the HSQC spectra in the aliphatic chain region confirmed the formation of sulfate esters (Supplementary Figure S3). Additionally, the increased methoxyl group content (Table 3) points to concurrent methylation, likely via the transfer of a methyl group from the MeSO_4_ anion.

Importantly, in this environment with low-basicity anions, the formation of NHC from the bmim cation is suppressed, explaining the negligible amount of covalently bound cations (Table 5). Thus, in this system, the cation plays a secondary structural role, whereas the chemical transformation is dictated by the anion.

### 3.4. Implications for Biorefining and Future Perspectives

Our findings have serious implications for the development of IL-based biorefining processes and reveal the following challenges and opportunities. The covalent incorporation of IL components into lignin challenges the concept of their “inertness” and creates significant complications for solvent recycling; the purification and reuse of ILs, particularly [bmim]OAc, after contact with lignin require the development of new energy-efficient methodologies. On the other hand, this controllable reactivity opens up unique opportunities for the targeted synthesis of functional lignin-based materials with tailored properties. The choice of IL allows for the selective production of fundamentally different products: (i) [bmim]OAc-modified lignin is a depolymerized, nitrogen-containing material. The presence of basic nitrogen heterocycles may impart the properties of a biocide, a chelating agent for metal ions, or a precursor for nitrogen-doped carbon materials and novel polyurethanes. (ii) [bmim]MeSO_4_-modified lignin is a condensed, sulfur-enriched polymer. Its potential applications include use as a phenolic substitute in adhesives and resins, a reinforcing additive in composites, or a feedstock for sulfur-containing carbon fibers and molecular sieves.

Furthermore, expanding the scope to include more acidic ionic liquids, such as [bmim]HSO_4_, would allow for a more comprehensive understanding of how the acid strength of the anion influences the balance between condensation and functionalization pathways, building upon the trends observed here with [bmim]MeSO_4_.

Thus, this research shifts the use of imidazolium ILs in lignin processing from the paradigm of “passive dissolution” to the paradigm of “active chemical design”. Future research should focus on finding ILs with controllable reactivity that allow for targeted modification while maintaining the possibility of efficient recycling.

In summary, the key advantage of this work is the comprehensive multitechnique approach that directly correlates macroscopic changes in lignin properties (M_w_, functional groups) with molecular-level evidence of covalent modification, providing an unambiguous demonstration of anion-dependent reactivity. The main limitation is the focus on a model lignin substrate (spruce dioxane lignin) and a specific set of ILs, which calls for future studies on more complex, native lignocellulosic substrates and a broader range of IL structures to assess the general applicability of these findings fully.

## 4. Materials and Methods

### 4.1. Materials

The ionic liquids 1-butyl-3-methylimidazolium acetate ([bmim]OAc), chloride ([bmim]Cl), and methyl sulfate ([bmim]MeSO_4_) with purities of >95% (BASF quality) were purchased from Sigma–Aldrich (Steinheim, Germany). The derivatization reagent for NMR analysis of hydroxy groups, 2-chloro-4,4,5,5-tetramethyl-1,3,2-dioxophospholane, was purchased from the same supplier. Deuterated solvents (pyridine-*d*_5_, chloroform-*d*_1_, and DMSO-*d*_6_) were supplied by Deutero GmbH (Kastellaun, Germany) and had a purity of >99%. Analytical grade (purum p.a.) 1,4-dioxane and hydrochloric acid were obtained from Chimmed (Moscow, Russia). HPLC gradient grade acetonitrile (Greenvan, St.-Peterburg, Russia) was used for chromatographic separations. All aqueous solutions were prepared using Type I Milli-Q deionized water with a resistivity of 18.2 MΩ∙cm.

### 4.2. Lignin Isolation and Treatment Procedures

A low-altered dioxane lignin (DL) sample was isolated from *Picea Abies* (Norway spruce) wood via a commonly accepted Pepper method [41] with slight modifications. The starting wood flour (particle size < 0.2 mm) had the following chemical composition (on a dry wood basis): 27.2% Klason lignin and 70.5% total polysaccharides, of which 47.2% was cellulose, as determined via the common Kurschner method. Briefly, the wood flour was initially deresinated with acetone in a Soxhlet apparatus and then extracted with 0.1 M HCl in 90% aqueous 1,4-dioxane at 80 °C for 2 h under a nitrogen atmosphere. The resulting solution was neutralized and concentrated by evaporation under reduced pressure. Lignin was precipitated by adding excess water, washed, filtered, and vacuum-dried at 40 °C for 24 h. The yield of DL was 10% on the basis of dry wood weight.

Ionic liquid lignin (IL-DL) samples were prepared by treating DL (100 mg) with 1 g of IL in airtight 15 mL glass vials at 80, 100, 120, and 150 °C for 24 h. After treatment, the IL-DL preparations were isolated by resuspension in a 20-fold excess of water, followed by filtration, thorough washing with water, and vacuum drying. All the treatments and subsequent IL-DL analyses were performed in triplicate.

The reaction time of 24 h was selected on the basis of preliminary kinetic data (Tables S1 and S2), which indicated that the incorporation of nitrogen (for [bmim]OAc) and sulfur (for [bmim]MeSO_4_) reached near-plateau or significant levels at this time, especially at the highest treatment temperatures, suggesting that major transformation trends were established.

### 4.3. Analytical Methods

Elemental analysis (CHNS) was carried out by catalytic combustion on a EuroEA-3000 CHNS analyzer (EuroVector, Pavia, Italy). The oxygen content was calculated by the difference.

The weight-average (M_w_) and number-average (M_n_) molecular weights and molecular weight distributions of the obtained lignin preparations were determined via high-performance size-exclusion chromatography on an LC-20 HPLC system (Shimadzu, Kyoto, Japan) consisting of an LC-20AD chromatographic pump, a SIL-20A autosampler, a CTO-20A column thermostat, and an SPD-20A spectrophotometric detector (275 nm). The size-exclusion separations were performed on an MCX column (300 × 8 mm, pore size of 1000 Å) for anionic polymer analysis (PSS, Meinz, Germany) using 0.1 M NaOH as the mobile phase and a sample solvent. The system was calibrated in the range of 0.3–680 kDa using a set of sodium polystyrene sulfonate standards.

NMR spectra were recorded on an AVANCE III spectrometer (Bruker Biospin, Ettlingen, Germany) with an operating frequency of 600 MHz (^1^H) via TopSpin ver. 3.2 instrument control software. The hydroxyl group content was quantified via ^31^P NMR after derivatization with 2-chloro-4,4,5,5-tetramethyl-1,3,2-dioxophospholane in a pyridine-*d*_5_/chloroform-*d*_1_ mixture [42]. For the 2D NMR (HSQC) experiments, approximately 30 mg of the lignin sample was dissolved in 0.6 mL of DMSO-*d*_6_. The spectra were recorded at 298 K with a 12 μs pulse duration, 0.9 s acquisition time, 0.11 s pulse delay, and 65,000 accumulations. An ACD/Labs NMR Workbook suite software, ver. 2019 (ACD/Labs, Toronto, ON, Canada), was used to process and interpret the 2D NMR spectroscopic data.

High-performance liquid chromatography–high-resolution mass spectrometry (HPLC-HRMS) analyses were carried out via an LC-30 Nexera HPLC system (Shimadzu, Kyoto, Japan) coupled with an Orbitrap Q Exactive Plus hybrid (quadrupole–orbital ion trap) mass spectrometer (Thermo Scientific, Waltham, MA, USA) with an electrospray (ESI) ion source. Chromatographic separation was achieved on a reversed-phase Nucleodur PFP column (150 × 3 mm, 1.8 μm) with a pentafluorophenyl-grafted silica sorbent (Macherey-Nagel, Duren, Germany) at 40 °C. A gradient elution with a water/acetonitrile mixture (0.45 mL/min total flow) was programmed as follows: 0–2 min—% CH_3_CN, 2–33 min—a linear ramp to 70%, 33–35 min—100%, 36–43 min—equilibrating the column with 20% CH_3_CN. The mass spectrometry parameters used were as follows: positive ion detection mode (ESI+), sheath and aux gas pressures of 25 and 10 psi, respectively; transfer capillary temperature, 320 °C; source temperature, 200 °C; spray voltage, 3.8 kV; and S-lens RF level, 55%. Full MS scans in the range of *m*/*z* 150–1000 followed by data-dependent MS/MS scans (*m*/*z* 50–1000) with a collision energy of 30 eV (higher-energy collision-induced dissociation, HCD) were used for nontargeted screening of lignin oligomers. The mass analyzer resolving power was set to 70,000 and 30,000 (FWHM, at *m*/*z* 200) in the MS and MS/MS regimes, respectively. An automatic gain control with a C-Trap target filling value of 5∙10^5^ ions was used. Data acquisition was performed via Xcalibur instrument control software (ver. 3.1) (Thermo Scientific, Waltham, MA, USA). Freestyle (ver. 1.8) and Compound Discoverer (ver. 2.1) software (Thermo Scientific, Waltham, MA, USA) were used for HPLC-HRMS data mining and structure elucidation. The accurate mass-based elemental compositions of the detected lignin oligomers were calculated by applying the following constraints on the number of atoms and other parameters: C, H, O—6–100; S, N—0–10; ring and double bond equivalent (DBE, unsaturation degree)—4–30; charge state—+1; and *m*/*z* tolerance—3 ppm. The mass scale was calibrated daily according to the manufacturer’s recommendations.

## 5. Conclusions

This study unequivocally demonstrates that 1-butyl-3-methylimidazolium-based ionic liquids are not inert solvents but rather active chemical agents that dictate the transformation pathways of lignin through distinct, anion-dependent mechanisms.

The nature of the IL anion determines fundamentally different modification routes. The acidic methyl sulfate anion ([bmim]MeSO_4_) promotes acid-catalyzed condensation and anion-driven functionalization, resulting in a significant increase in molecular weight (up to 15.2 kDa) and intense sulfur incorporation (up to 9.9%). In contrast, the highly basic acetate anion ([bmim]OAc) directs the process toward depolymerization and covalent cation grafting, as evidenced by a decrease in the M_w_ and high nitrogen content (up to 6.5%).

For [bmim]OAc, it was conclusively proven that the covalent grafting of the bmim cation onto lignin (up to 11 cations per 100 aromatic units) proceeds via a nucleophilic addition mechanism involving an N-heterocyclic carbene derived in situ from the cation. Key evidence includes the complete elimination of aldehyde groups, 2D NMR data, and direct identification of hybrid N-containing oligomers via HPLC-HRMS. This “capping” process effectively prevents the recondensation of reactive lignin fragments.

The chloride anion exhibits properties similar to but less pronounced than those of [bmim]MeSO_4_, primarily inducing acid-catalyzed transformations without significant covalent incorporation of the IL components, underscoring the crucial role of acetate basicity in cation activation.

These findings challenge the perception of ILs as “green” and inert solvents, as the covalent incorporation of their components into biopolymers poses serious challenges for IL recycling. However, this controllable reactivity also opens new avenues for the targeted synthesis of functional lignin-based materials with tailored properties—from condensed sulfur-containing polymers to depolymerized nitrogen-containing oligomers—thereby expanding their potential for practical application.

This study has several limitations. The long-term stability of the obtained IL–lignin adducts and the potential for multiple recycling cycles of ionic liquids, particularly the reactive [bmim]OAc, were not investigated and represent crucial directions for future research. Furthermore, expanding the study to include a wider range of ILs with varying acidities and basicities could provide a more comprehensive understanding of the structure–reactivity relationships. Another limitation of this study is that a detailed kinetic analysis was not performed for all structural parameters; however, the time-dependent elemental analysis supported the selection of the 24 h timepoint for capturing the dominant transformation pathways.

An important aspect not addressed in this study is the state and recyclability of the ionic liquids after the reaction. The covalent incorporation of IL components into lignin, particularly in the case of [bmim]OAc, suggests that the IL structure is not fully preserved and that its regeneration for repeated cycles may be challenging. Investigating the efficiency of IL recycling and the development of purification protocols constitute critical directions for future applied research.

Thus, this work shifts the use of imidazolium ILs in lignin processing from the paradigm of passive dissolution to the paradigm of active chemical design, where the choice of IL allows for deliberate engineering of the final product’s structure and properties.

## Figures and Tables

**Table 1:** Molecular Weight Distribution of Dioxane Lignin upon Thermal Treatment in 1-butyl-3-methylimidazolium Ionic Liquids.

IL	T, °C	M_n_, kDa	M_w_, kDa	PDI ^1^ (M_w_/M_n_)	Content of the fraction, %		
					<1 kDa	1–20 kDa	>20 kDa
[bmim]OAc	80	1.5	7.7	5.1	7	88	5
	100	1.4	7.7	5.5	7	88	5
	120	1.2	5.7	4.8	10	88	2
	150	1.2	3.6	3.0	13	86	1
[bmim]Cl	80	2.2	10.9	5.0	4	85	11
	100	2.3	11.1	4.8	5	84	11
	120	2.7	10.4	3.9	4	86	10
	150	2.5	7.8	3.1	4	88	8
[bmim]MeSO_4_	80	2.0	9.4	4.9	5	88	7
	100	2.2	9.0	4.5	4	90	6
	120	2.7	11.5	4.5	4	88	8
	150	3.4	15.2	4.4	2	84	14

^1^ Polydispersity index.

**Table 2:** Contents of hydroxyl and carboxyl groups in lignin after treatment with bmim-based ILs determined by ^31^P NMR.

IL	T, °C	Content, % (*w*/*w*)			
		-OH_total_	-OH_phen_	-OH_aliph_	-COOH
DL	-	9.1	3.3	5.8	0.1
[bmim]OAc	80	9.9	3.5	6.4	0.4
	100	9.7	3.9	5.8	0.5
	120	8.7	4.0	4.7	0.7
	150	8.6	5.1	3.5	1.4
[bmim]Cl	80	9.7	3.9	5.8	0.7
	100	9.0	3.7	5.3	0.7
	120	9.2	4.2	5.0	0.8
	150	10.4	4.3	6.1	0.2
[bmim]MeSO_4_	80	7.0	3.2	3.8	0.6
	100	6.4	3.3	3.1	0.6
	120	5.9	3.0	2.9	0.6
	150	3.7	2.4	1.3	0.2

**Table 3:** Contents of the main interunit linkages and methoxyl and aldehyde groups in the initial DL and lignin preparations after treatment with bmim-based ILs at 150 °C, as determined by 2D HSQC NMR.

Sample	Groups and Interunit Linkages Content, Number per 100 Aromatic Units				
	-OCH_3_	-C=O	β-O-4	α-O-4/β-5	α-O-γ/β-β
DL	96	10.4	21.4	7.4	3.5
IL-DL ([bmim]OAc)	79	0.0	4.1	1.1	2.3
IL-DL ([bmim]Cl)	88	1.6	3.6	4.4	3.4
IL-DL ([bmim]MeSO_4_)	106	3.0	0.0	8.4	1.1

**Table 4:** Elemental composition (CHNS/O) and heteroatom incorporation in lignin after thermal processing with ionic liquids.

Sample	T, °C	Elemental Composition, %				
		C	H	N	S	O
DL		62.6	7.3	0.0	0.0	30.1
IL-DL ([bmim]OAc)	80	63.9	7.9	2.4	0.0	25.8
	100	64.5	7.7	2.1	0.0	25.7
	120	65.1	8.0	3.3	0.0	24.7
	150	65.5	8.2	6.5	0.0	19.7
IL-DL ([bmim]MeSO_4_)	80	60.2	6.6	2.7	1.2	29.3
	100	59.2	6.8	3.6	1.9	28.5
	120	60.1	6.9	3.8	2.0	27.2
	150	58.3	5.2	4.7	9.9	21.9
IL-DL ([bmim]Cl)	80	64.7	6.0	1.0	0.0	28.3
	100	64.8	5.9	0.9	0.0	28.4
	120	64.9	5.9	0.9	0.0	28.3
	150	65.0	5.8	0.9	0.0	28.3

**Table 5:** ^1^H-^13^C HSQC NMR quantification of the IL-derived fragments in the IL-DL samples obtained at different treatment temperatures.

T, °C	Covalently Bound bmim, Number per 100 Aromatic Units		
	[bmim]OAc	[bmim]MeSO_4_	[bmim]Cl
80	2.9	3.2	3.8
100	4.0	2.4	1.8
120	4.6	1.0	2.3
150	10.8	0.5	4.5

**Table 6:** Major Nitrogen-Containing Compounds Identified by HPLC–HRMS from Lignin Treated with [bmim]OAc at 150 °C.

No. ^1^	Elemental Composition	RDB ^2^	Retention Time, min	*m*/*z*	Δ, ppm
1	[C_16_H_23_O_2_N_2_]^+^	6.5	3.32	275.1755	0.26
2	[C_17_H_25_O_3_N_2_]^+^	6.5	3.41	305.1860	0.10
3	[C_27_H_35_O_6_N_2_]^+^	11.5	7.15	483.2487	0.51
4	[C_25_H_29_O_5_N_2_]^+^	12.5	9.87	437.2072	0.33
5	[C_27_H_33_O_5_N_2_]^+^	12.5	12.07	465.2386	0.41
6	[C_28_H_35_O_6_N_2_]^+^	12.5	12.12	495.2491	0.24

^1^ Corresponds to the peak numbers in Figure 2. ^2^ Ring and double bond equivalent (unsaturation degree).

## Data Availability

The original contributions presented in this study are included in the article/Supplementary Materials. Further inquiries can be directed to the corresponding authors.

## Supplementary Materials

The following supporting information can be downloaded at: https://www.mdpi.com/article/10.3390/ijms262311627/s1.

## Abbreviations

The following abbreviations are used in this manuscript:

bmim	1-Butyl-3-methylimidazolium
DL	Dioxane lignin
ESI	Electrospray ionization
HPLC	High-performance liquid chromatography
HRMS	High-resolution mass spectrometry
HSQC	Heteronuclear single quantum coherence
IL	Ionic liquid
MWD	Molecular weight distribution
NHC	N-heterocyclic carbene
NMR	Nuclear magnetic resonance
PDI	Polydispersity index
RDB	Ring and double bond equivalent
TIC	Total ion current
XIC	Extracted ion current
