# Supplementary material for: Chemical Transformations of Lignin Under the Action of 1-Butyl-3-Methylimidazolium Ionic Liquids: Covalent Bonding and the Role of Anion
Source: Int J Mol Sci. 2025 Nov 30;26(23):11627. doi: 10.3390/ijms262311627 (PMC12691707; doi:10.3390/ijms262311627)
Supplement: Supplementary file 1 [file ijms-26-11627-s001.zip › ijms-4004012-supplementary.pdf]

Supplementary material

Chemical Transformations of Lignin Under the Action of 1-Butyl-3-Methylimidazolium Ionic Liquids: Covalent Bonding and the Role of Anion

Artyom V. Belesov *, Ilya I. Pikovskoi, Anna V. Faleva and Dmitry S. Kosyakov *

Core Facility Center 'Arktika', M.V. Lomonosov Northern (Arctic) Federal University, Arkhangelsk 163002, Russia

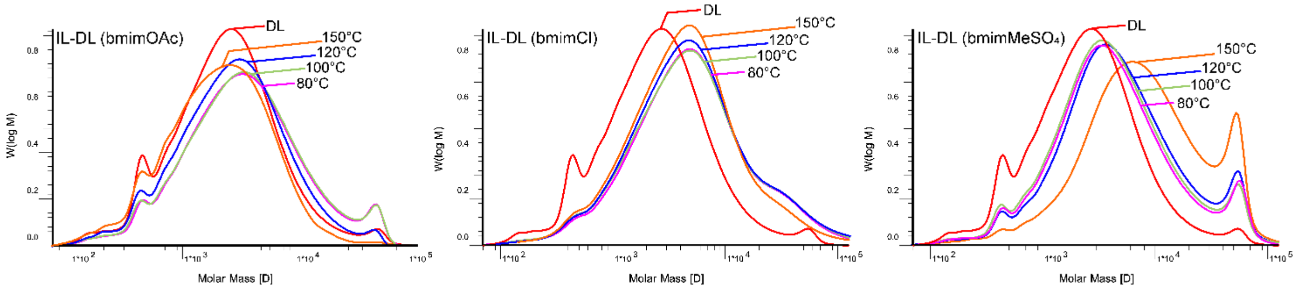

Figure S1. Evolution of molecular weight distributions of spruce dioxane lignin (DL) upon thermal treatment in 1-butyl-3-methylimidazolium ionic liquids (IL-DL).

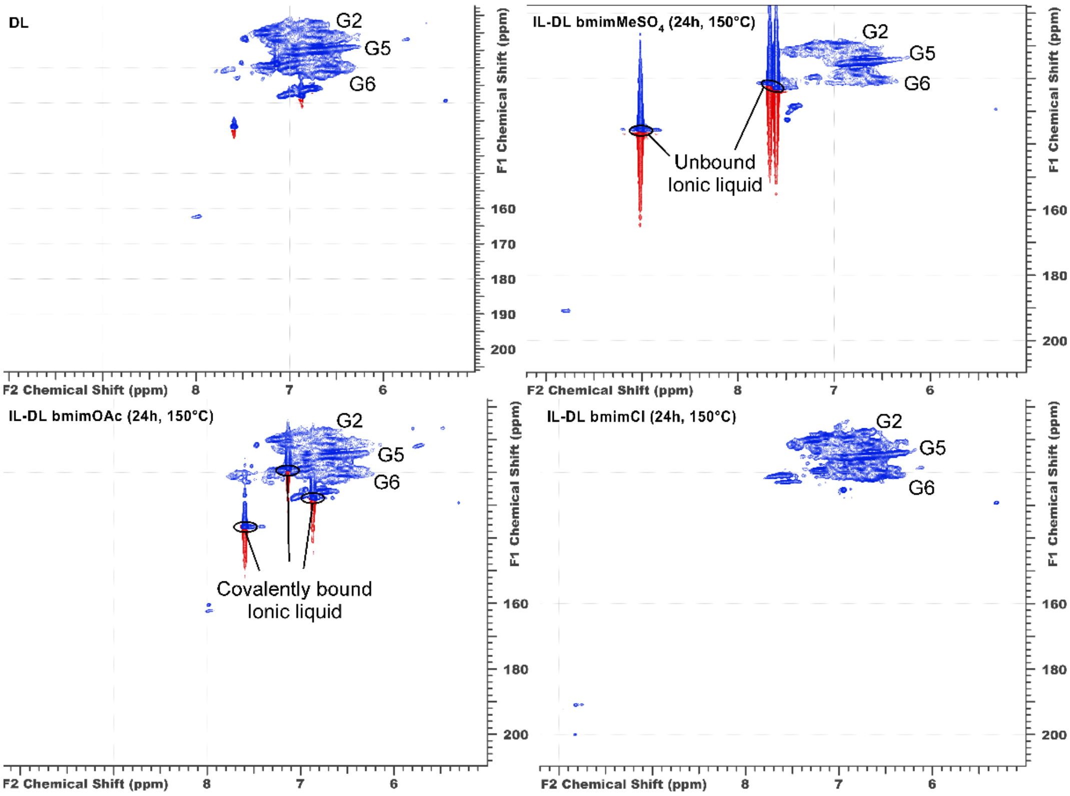

Figure S2. 2D ^{1}H– ^{13}C HSQC NMR spectra (aromatic region) of lignin after treatment with [bmim]-based ILs at 150 °C.

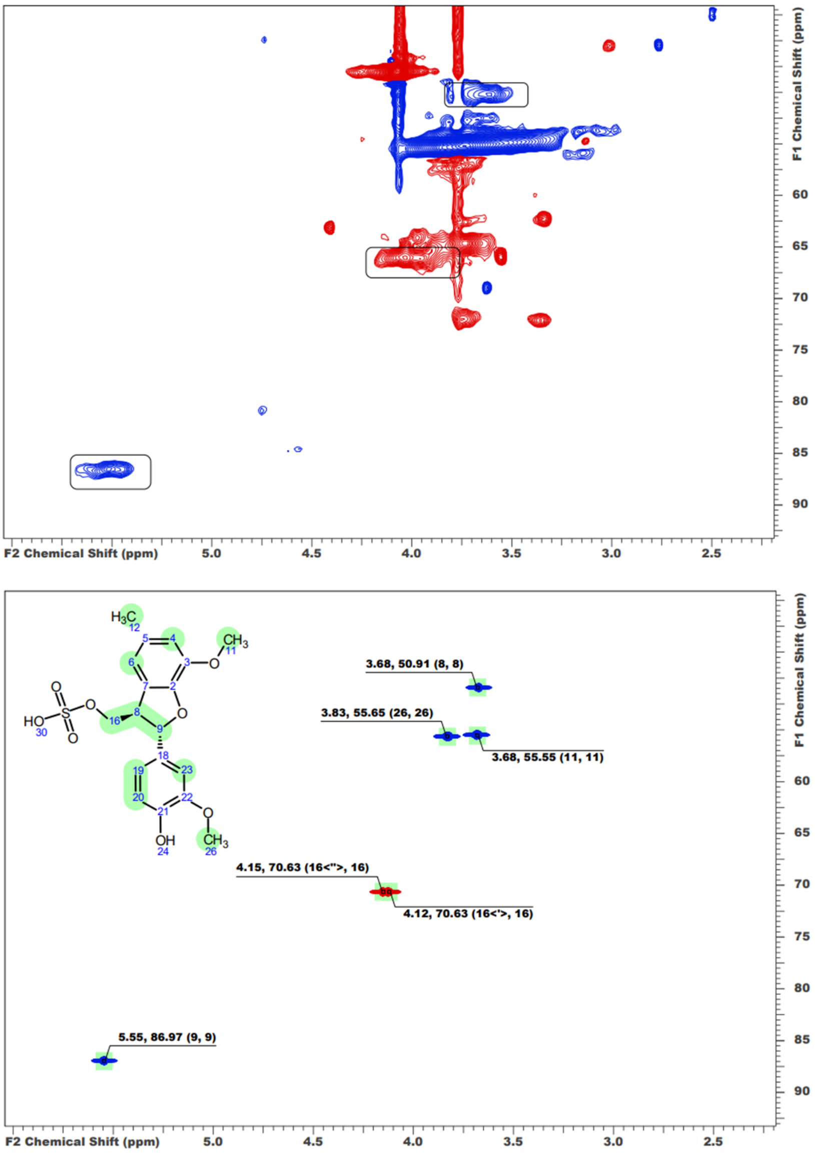

Figure S3. Assignment of key structures in the aliphatic region of the 2D HSQC spectrum for Lignin treated with [bmim]MeSO4.

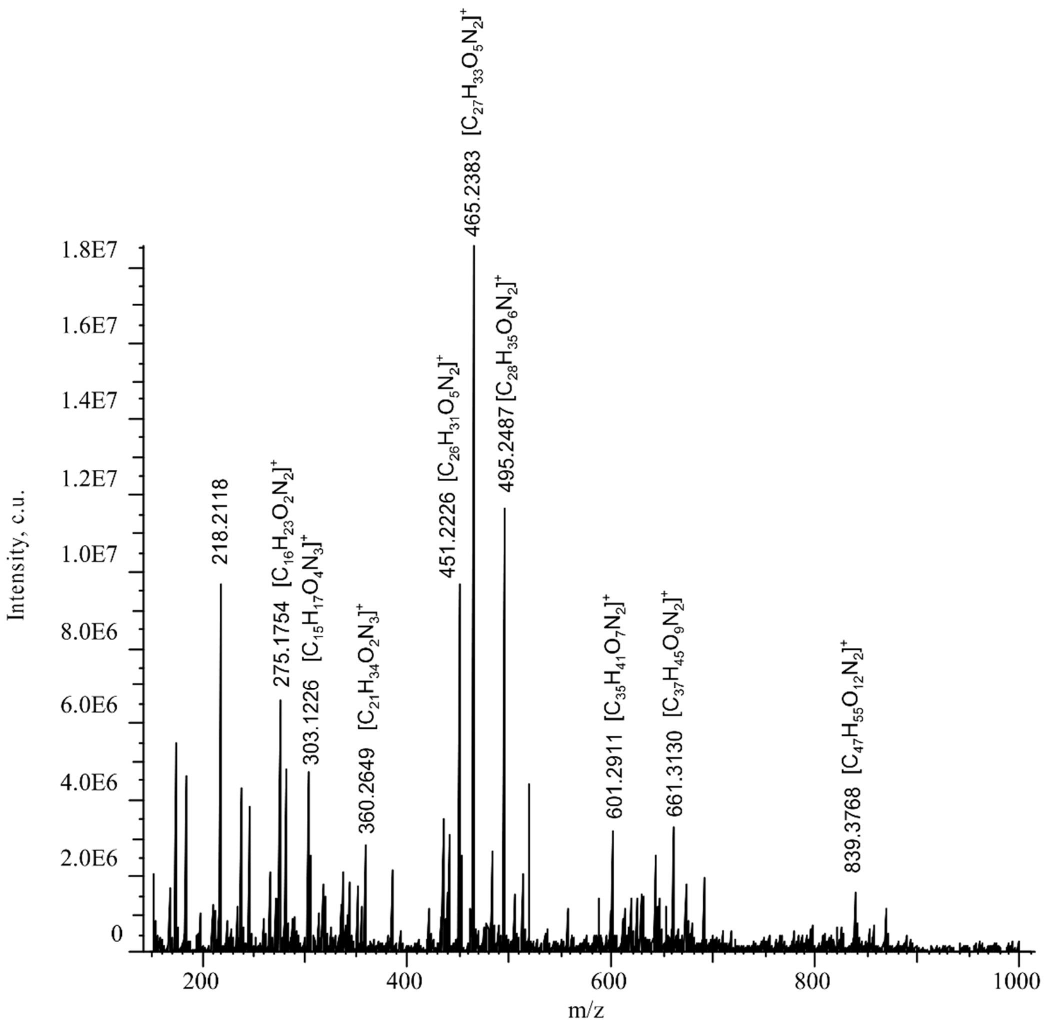

Figure S4. High-resolution ESI-Orbitrap mass spectrum (positive ion mode) of the methanol-soluble fraction of lignin treated with [bmim]OAc at 150 °C.

Table S1. Nitrogen content in the composition of the obtained IL-DL preparations, depending on the treatment duration and temperature.

Processing conditions		Nitrogen content, %		
		[bmim]OAc	[bmim]$MeSO_4$	[bmim]Cl
80 °C	2 h	1.1	1.6	0.8
	4 h	1.2	1.2	2.0
	8 h	1.5	0.7	1.0
	24 h	2.4	2.7	1.0
100 °C	2 h	1.1	1.9	0.7
	4 h	1.2	2.0	1.5
	8 h	1.3	2.2	0.9
	24 h	2.1	3.6	0.9
120 °C	2 h	1.2	2.2	0.6
	4 h	1.3	2.8	1.4
	8 h	1.3	3.4	0.8
	24 h	3.3	3.8	0.9
140 °C	2 h	2.0	2.7	0.6
	4 h	2.3	3.4	0.8
	8 h	4.0	3.8	0.7
	24 h	6.5	4.7	0.9

Table S2. Sulfur content in the composition of the obtained IL-DL preparations depending on the [bmim]$MeSO_4$ treatment duration and temperature.

Processing conditions		Sulfur content, %
80 °C	2 h	0.0
	4 h	0.3
	8 h	0.0
	24 h	1.2
100 °C	2 h	0.7
	4 h	0.9
	8 h	1.1
	24 h	1.9
120 °C	2 h	2.1
	4 h	1.7
	8 h	2.9
	24 h	2.0
140 °C	2 h	2.7
	4 h	2.5
	8 h	9.4
	24 h	9.9
